# Initial experience of dedicated breast PET imaging of ER+ breast cancers using [F-18]fluoroestradiol

**DOI:** 10.1038/s41523-019-0107-9

**Published:** 2019-04-16

**Authors:** Ella F. Jones, Kimberly M. Ray, Wen Li, Amy J. Chien, Rita A. Mukhtar, Laura J. Esserman, Benjamin L. Franc, Youngho Seo, Miguel H. Pampaloni, Bonnie N. Joe, Nola M. Hylton

**Affiliations:** 1 Department of Radiology and Biomedical Imaging, University of California, San Francisco, CA USA; 2 Helen Diller Family Comprehensive Cancer Center, University of California, San Francisco, CA USA; 3 Department of Surgery, Helen Diller Family Comprehensive Cancer Center, University of California, San Francisco, CA USA

## Abstract

Dedicated breast positron emission tomography (dbPET) is an emerging technology with high sensitivity and spatial resolution that enables detection of sub-centimeter lesions and depiction of intratumoral heterogeneity. In this study, we report our initial experience with dbPET using [F-18]fluoroestradiol (FES) in assessing ER+ primary breast cancers. Six patients with >90% ER+ and HER2− breast cancers were imaged with dbPET and breast MRI. Two patients had ILC, three had IDC, and one had an unknown primary tumor. One ILC patient was treated with letrozole, and another patient with IDC was treated with neoadjuvant chemotherapy without endocrine treatment. In this small cohort, we observed FES uptake in ER+ primary breast tumors with specificity to ER demonstrated in a case with tamoxifen blockade. FES uptake in ILC had a diffused pattern compared to the distinct circumscribed pattern in IDC. In evaluating treatment response, the reduction of SUV_max_ was observed with residual disease in an ILC patient treated with letrozole, and an IDC patient treated with chemotherapy. Future study is critical to understand the change in FES SUV_max_ after endocrine therapy and to consider other tracer uptake metrics with SUV_max_ to describe ER-rich breast cancer. Limitations include variations of FES uptake in different ER+ breast cancer diseases and exclusion of posterior tissues and axillary regions. However, FES-dbPET has a high potential for clinical utility, especially in measuring response to neoadjuvant endocrine treatment. Further development to improve the field of view and studies with a larger cohort of ER+ breast cancer patients are warranted.

## Introduction

Advances in our understanding of breast cancer biology have provided an improved ability to provide patients with prognostic information and treat their disease with precisely targeted therapy regimens. One of the most dramatic of these advances has been in endocrine therapy whereby the estrogen-based signaling for tumor growth is interrupted in estrogen receptor positive (ER+) breast cancer patients. For over three decades, clinical evidence has suggested that ER+ tumors are responsive to endocrine treatment.^1,2^ However, not all ER+ tumors derive the same benefit from endocrine therapy, and some patients with ER+ breast cancer will have improved outcomes with additional systemic therapy such as chemotherapy. Genomic assays such as Oncotype DX™ (Genomic Health, CA, USA)^3,4^ and MammaPrint™ (Agendia BV, The Netherlands)^5,6^ can help identify which patients can be safely treated without chemotherapy and estimate the potential benefit of chemotherapy. Despite this, there is still uncertainty in the management of many women with ER+ breast cancer.

Intratumoral heterogeneity of ER expression in some breast tumors poses a dilemma; sampling by biopsy may produce varying results with different outcomes in overall tumor assessment.^7^ In vivo non-invasive whole-tumor assessment could theoretically decrease the uncertainty associated with biopsy sampling. Non-invasive molecular imaging has served as an in vivo assay to assess for distant disease in breast cancer patients, and [^18^F]fluorodeoxyglucose (FDG) positron emission tomography (PET) imaging plays a crucial role in evaluating for metastatic disease in breast cancer staging. In assessing primary tumors, however, the standard whole-body PET/CT is hampered by limited spatial resolution that results in significant partial volume effect that complicates quantification of small lesions. In addition, when whole-body PET/CT is performed with the patient in a supine position, the breast volume is naturally collapsed and depiction of the primary tumor is not ideal. Dedicated breast PET (dbPET) is an emerging PET technology specially designed for breast imaging. Compared to the whole-body PET/CT using FDG as a tracer, dbPET with the patient in a prone position has demonstrated a higher sensitivity^8^ to detect sub-centimeter lesions and a higher spatial resolution to depict intratumoral heterogeneity^9^ in clinical studies. In our own work, we have also demonstrated the use of dbPET with FDG to evaluate the primary tumors in a breast cancer patient. At high sensitivity, dbPET can capture the early response to neoadjuvant chemotherapy in primary tumors and reveal functional changes that precede anatomic changes at magnetic resonance imaging (MRI).^10^

In addition to evaluating tumor glucose metabolism by FDG, PET offers other functional information by the use of ligands labeled with positron-emitting radionuclides. Estradiol, a form of estrogen, is a potent agonist for ER. ^18^F-fluoroestradiol (FES) has been developed specifically to image ER expression in breast cancers.^11,12^ Recent clinical studies have shown that FES-PET has a high overall sensitivity (84%) and specificity (98%) in assessing the ER status in breast cancers,^13^ and FES uptake has clear potential to guide therapy selection and to predict endocrine treatment response.^14^ In this study, we further evaluate the use of the dbPET with FES to assess the ER expression and its change in response to treatment in the ER+ breast cancer subtype.

## Results

### Primary tumor characterization

**Fig. 1:**
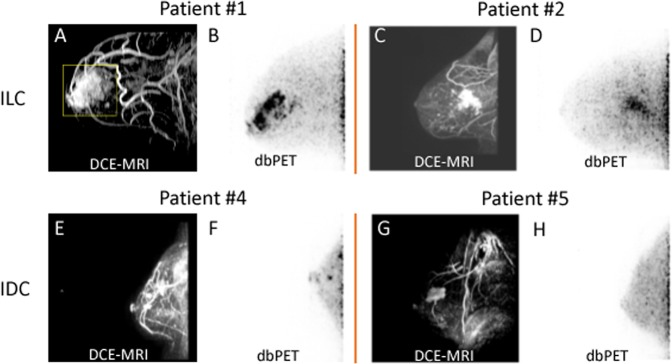
Examples of FES uptake in >90% ER+/HER2− invasive lobular carcinomas (ILC, top panel) and invasive ductal carcinomas (IDC, bottom panel). Top—**a** Patient #1—a 61-year-old female patient with grade 2 ILC in her right breast. MRI showed contrast enhancement spanning 6.7 cm. **b** FES-dbPET showed an SUV_max_ at 15.83 and total uptake volume at 15.72 cm^3^. **c** Patient #2—a 64-year-old female patient with grade 2 ILC in her right breast. MRI showed an irregularly shaped mass with spiculated margins associating with non-mass enhancement of 5.3 cm. **d** The corresponding FES-dbPET showed a SUV_max_ at 22.42 and total uptake volume at 35.63 cm^3^. Bottom—**e** Patient #4—a 63-year-old female patient with grade 2 IDC in her left breast. MRI showed a 0.9 cm confirmed IDC and a 1.3 cm irregular mass with posterior depth. **f** FES-dbPET showed a SUV_max_ at 7.44 corresponding to the 0.9 cm anterior mass but missed the posterior mass that was close to the chest wall. **g** Patient #5—a 33-year-old female patient with grade 2 IDC in her right breast. MRI showed an enhancing mass with spiculated margin spanning 3.4 cm. **h** The patient was treated with tomoxifen for fertility preservation and stopped 4 days prior to FES-dbPET imaging. The corresponding FES-dbPET showed no FES uptake that was consistent with ER blockade

Six patients diagnosed with >90% ER+ and human epidermal growth factor receptor-2 negative (HER2−) breast cancer by immunohistochemistry (IHC) and fluorescence in situ hybridization (FISH) were imaged. Patient and tumor characteristics are summarized in Table 1, and the FES-dbPET imaging results are summarized in Table 2. Patient ages ranged from 33 to 64. Patients #1 and 2 had infiltrating invasive lobular carcinomas (ILC) with no lymphadenopathy or distant metastasis by ultrasound or bone scan. Their tumor measured respectively up to 6.7 and 5.3 cm by dynamic contrast-enhanced MRI (DCE-MRI). Their corresponding maximum FES standardized uptake value (SUV_max_) was 15.83 and 22.42 with total FES uptake volume at 15.72 and 35.63 cm^3^, respectively (Fig. 1a–d). The remaining patients were presented with locally advanced diseases, three with invasive ductal carcinomas (IDC) and one with an unknown primary tumor. No lymphadenopathy or distant metastasis was noted in IDC patients by whole-body FDG-PET/CT or clinical exams. Patient #3 had a primary tumor measuring 2.8 cm by DCE-MRI and SUV_max_ of 6.44 by whole-body FDG-PET/CT. The primary ER+ tumor was re-demonstrated with FES-dbPET SUV_max_ at 13.02 and total uptake volume at 12.60 cm^3^. The MRI of patient #4 showed a 0.9 cm IDC and a 1.3 cm irregular mass. Her FES-dbPET showed an SUV_max_ at 7.44 (uptake volume of 0.22 cm^3^) corresponding to the 0.9 cm anterior mass but the more posterior disease foci seen on MRI were excluded from the field of view of dbPET (Fig. 1e, f). Patient #5 demonstrated the absence of FES uptake in her 3.4 cm IDC, which was due to estrogen receptor blockade from recent administration of tamoxifen for a fertility preservation procedure (Fig. 1g, h). The final patient (#6) had metastatic cervical and axillary lymphadenopathy secondary to a breast primary that was occult on mammography and MRI. Her whole-body FDG-PET/CT showed multiple hypermetabolic cervical lymph nodes measuring up to 2.2 cm with SUV_max_ of 6.0 and axillary lymph nodes measuring up to 2.7 cm with SUV_max_ of 4.5. Her bilateral breast tissues were heterogeneously FDG-avid with no hypermetabolic mass. FES-dbPET also showed no corresponding uptake in the breast bilaterally.

**Table 1:** Patient and tumor characteristics

Patient	Age	Disease	ER status	HER 2 status (FISH^a^)	Genetic assay score	Tumor grade	Stage	Neoadjuvant therapy
**1**	61	ILC	95%	HER2:CEP17 = 1.1 HER2_ave_ = 1.9	MammaPrint Low	2	ypT2N1a	Letrozole
**2**	64	ILC	>95%	HER2:CEP17 = 1.2 HER2_ave_ = 3.2	MammaPrint Low	2	pT3N1a	None
**3**	49	IDC	100%	HER2:CEP17 = 1.1 HER2_ave_ = 2.0	MammaPrint High	2	ypT2N1mic	Chemotherapy w/o endocrine therapy
**4**	63	IDC	90%	HER2:CEP17 = 1.1 HER2_ave_ = 1.9	Oncotype Low	2	pT2	None
**5**	33	IDC	>95%	HER2:CEP17 = 2.0 HER2_ave_ = 2.9	MammaPrint High	2	ypT2N1a	Chemotherapy w/o endocrine therapy
**6**	34	Unknown	>90%	HER2:CEP17 = 1.3 HER2_ave_ = 5.1	None	3	Denovo metastatic unknown primary	None

^a^HER2 gene expression amplification by FISH:

Not amplified: HER2:CEP17 ratio <2.0 AND HER2_ave_ <4.0 signals per cell

Equivocal: HER2:CEP17 ratio <2.0 AND HER2_ave_ ≥4.0 and <6.0 signals per cell

Amplification: HER2:CEP17 ratio ≥2.0 OR HER2_ave_ ≥6.0 signals per cell

**Table 2:** Summary of FES-dbPET imaging results

Patient	SUV_max_	SUL_max_	Tumor-normal ratio	FES uptake volume (cm^3^)
1	15.83 (V1)	9.32 (V1)	4.81 (V1)	15.72 (V1)
	6.11 (V2)^a^	3.58 (V2)^a^	2.55(V2)^a^	0.37 (V2)^a^
2	22.42	15.65	4.3	35.63
3	13.02 (V1)	9.08 (V1)	8.04 (V1)	12.60 (V1)
	16.26 (V2)^b^	11.61 (V2)^b^	8.74 (V2)^b^	6.97 (V2)^b^
4	7.44	5.67	2.52	0.22
5	No FES uptake detected due to the prior fertility preservation treatment with tamoxifen			
6	No FES uptake detected			

^a^Patient FES-dbPET follow-up scan after 2 months of letrozole. V1 = baseline; V2 = follow-up

^b^Patient FES-dbPET follow-up scan after 3 weeks of chemotherapy without endocrine treatment. V1 = baseline; V2 = follow-up

### Treatment response assessment

**Fig. 2:**
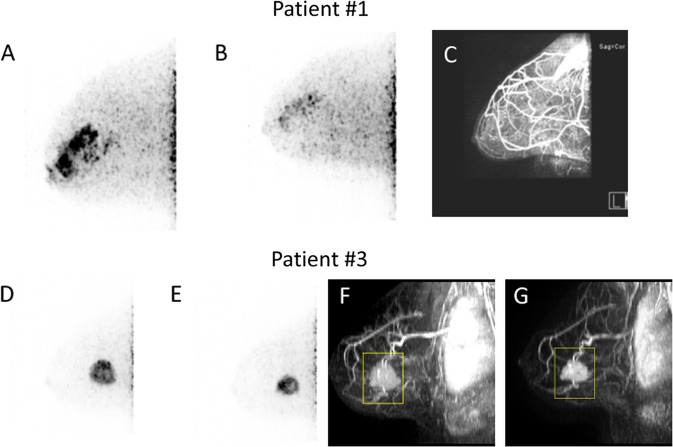
Examples of FES-dbPET for assessing treatment response. Top patient #1 with ILC—**a** FES-dbPET imaging at baseline with a SUV_max_ at 15.83 and total uptake volume at 15.72 cm^3^. **b** FES-dbPET of the same patient after 2 months of treatment with letrozole showing a SUV_max_ at 6.11 and total uptake volume at 0.37 cm^3^. **c** DCE-MRI after 3 months of treatment with letrozole, confirming the favorable response with no residual disease but with significant background enhancement. Bottom patient #3 with IDC—**d** A 49-year-old female patient with grade 2 IDC in her left breast. FES-dbPET imaging at baseline showed a SUV_max_ at 13.02 and total uptake volume at 12.60 cm^3^. **e** FES-dbPET of the same patient after 3 weeks of treatment with chemotherapy without endocrine therapy had a SUV_max_ at 16.26 but a reduced total uptake volume at 6.97 cm^3^ (45% reduction). **f**, **g** DCE-MRI at baseline and after 3 weeks of treatment, confirming the tumor size reduction by 40%

FES-dbPET was also used to monitor treatment response in Patient #1 and # 3. Patient #1 was imaged after 2 months of neoadjuvant endocrine therapy (inhibition of estrogen production with letrozole). Her FES uptake measured by SUV_max_ decreased from 15.83 to 6.11 and total uptake volume reduced from 15.72 to 0.37 cm^3^. Her follow-up DCE-MRI showed tumor reduction in the extent of non-mass enhancement with significant background enhancement (Fig. 2a–c). Surgical pathology showed 5 cm of residual ILC with Ki67 of <1% (reduced from 30% at pre-treatment) and tumor cellularity of 25% (pre-treatment cellularity not available).

Patient #3 participated in a neoadjuvant treatment trial and was treated with neoadjuvant chemotherapy without endocrine therapy. After three weekly cycles, her total FES uptake volume dropped from 12.60 to 8.73 cm^3^. Her DCE-MRI at the corresponding time point also confirmed a reduction in functional tumor volume (FTV)^15^ from 10.50 to 6.26 cm^3^ (Fig. 2d–f). However, the FES SUV_max_ showed an increase from 13.02 to 16.26. Surgical pathology showed 2.1 cm residual of grade 2 IDC with Ki67 of <1% (reduced from 20% at pre-treatment) and tumor cellularity of 5% (pre-treatment cellularity not available).

## Discussion

ER+ breast cancer presents a unique set of challenges for determining optimal treatment approaches. There is increased recognition that not all ER+ breast cancers benefit from chemotherapy and that there may be a subset of ER+ breast cancers that can be spared cytotoxic drugs and can be treated effectively using endocrine therapies alone. In the neoadjuvant treatment setting, imaging plays a critical role in non-invasively assessing the response of the intact primary tumor to targeted systemic therapies. Treatment-induced change in the primary tumor can serve as a surrogate marker for the effect of treatment. Thus, imaging evaluation of the primary tumor during treatment can provide important prognostic and predictive information.^16,17^ Db-PET imaging with FES as a tracer presents a new opportunity to develop an imaging marker for ER+ breast cancer. With the high overall sensitivity and specificity to ER, this new breast imaging tool may holistically inform the whole tumor ER functionality and provide valuable information to guide therapy selection. Herein, we report our effort to evaluate the FES-dbPET technology for enabling a more precise characterization of ER+ primary breast tumor and its response to neoadjuvant treatment.

In this small cohort, we observed variation in FES uptake even though all patients were >90% ER+/HER2− as determined by IHC and FISH. Variability of FES binding was also observed in another study with 91 ER+ breast cancer patients with an intraclass correlation coefficient of 0.6.^18^ The FES uptake variation may be attributed in part by the histologic characteristic of the breast cancer. At high resolution, dbPET was able to capture the FES uptake pattern in two ILC patients, showing a poorly circumscribed diffuse pattern (Fig. 1) that is consistent with the low tumor cell density and lack of desmoplastic stromal reaction characteristics.^19^ In contrary, the strong FES uptake in IDC was focal and well-defined, reflecting the high cellularity structurally similar phenotype.^20^

Imaging response to treatment was evaluated in two patients who underwent neoadjuvant therapy—one with letrozole, and one with chemotherapy. Letrozole is an aromatase inhibitor that prevents the activation of the ER by inhibiting the conversion of androgens to estrogens. Clinical studies^21,22^ have shown a clear benefit of letrozole over tamoxifen in treating ER+ breast cancers in postmenopausal women. In our case, we observed a reduction of FES uptake in a postmenopausal patient with ILC after two months of treatment, while surgical excision showed significant residual ILC. Given this patient had a marked improvement on the post-treatment MRI, these findings likely reflect that the patient might have started with a significant burden of disease that was unappreciated by currently available imaging techniques. Albeit incomplete, the reduction in MRI size, Ki67, and FES uptake are all consistent with response to therapy. An alternative explanation is that the reduction in FES uptake reflects non-functional ER in the remaining lobular disease, which could imply endocrine therapy resistance. Future study is critical to understand the significance of the change in FES uptake after endocrine therapy, particularly in ILCs that are treated primarily with endocrine agents.

Another patient with IDC was treated with chemotherapy without endocrine treatment. The FES SUV_max_ increased by 25% after three weekly cycles of treatment, but the FES uptake volume dropped by 31% and was confirmed by DCE-MRI. The partial response to chemotherapy was reflected in surgical pathology, which showed residual disease but low tumor cellularity. While SUV_max_ is a well-accepted metric for tracer uptake in primary tumors and metastatic diseases, it can be potentially biased with high variance depending on the noise level of the image.^23^ At high resolution, dbPET uses a smaller pixel size (0.5 mm vs. 3–5 mm in whole-body PET/CT) that may contribute to more noise and higher variance. In addition, SUV_max_ represents the maximum value for a lesion based on a single voxel, it may not adequately account for the binding of FES to the ER, and hence the overall ER functionality. In a randomized study of FES-PET as a predictor of response to neoadjuvent treatment in ER+ postmenopausal women,^24^ there was no correlation between baseline FES SUV_max_ and pathologic response in ER-rich breast cancer, suggesting other tracer uptake metrics should also be considered in conjunction with SUV_max_. In this study, we examined maximum SUV normalized by lean body mass (SUL), mean SUV (SUV_mean_) and tumor-to-normal tissue ratio, but none of them were able to describe the anatomical change observed by MRI. Future studies will include the change of SUV_max_ from baseline, volumetric measurement^25^ such as total uptake volume and textural features^26^ that reflect tumor heterogeneity in conjunction with SUV_max_, SUL, SUV_mean_, and tumor-to-normal tissue ratio as predictors of treatment response in a larger cohort.

The current version of the dbPET with the standard 200 mm aperture on the scan bed has limited posterior sensitivity near the chest wall. A new design described by O’Connor et al.^27^ has an enlarged aperture (220 mm), a thinner chest resting area and a flexible silicone sleeve to allow a significant gain of breast tissues near the chest wall within the detector field of view. However, the exclusion of the axillary lymph node remains a significant limitation of this technology. Modification of the scan bed with a larger flexible aperture, a new detector configuration with angular coverage and an improved image reconstruction algorithm may potentially mitigate the problem of the field of view and provide accurate quantification of the nodal signal. Further development in these areas is much needed.

In two patients, there was absence of FES uptake. One patient received tamoxifen prior to imaging, and the other had an unknown location of the primary breast tumor. Tamoxifen is a selective estrogen receptor modulator that binds to the ER and induces conformational changes^28^ that alter transcriptional activity. Tamoxifen will, therefore, affect the FES uptake. Some studies required the discontinuation of tamoxifen for 6–8 weeks prior to FES-PET imaging.^13,14^ In our case, the absence of FES uptake in patient #5 also reflects the specificity of FES binding to the ER.

FES uptake was not detected in the final patient (#6) with the unknown primary tumor that was occult from mammography and MRI. Whether the absence of uptake on FES is due to the location or size of the primary tumor, or whether this tumor is also occult on FES-PET imaging is unknown.

In conclusion, this feasibility study demonstrates that FES-dbPET imaging has potential as an imaging tool to characterize primary ER+ breast cancer and to guide therapy selection. Limitations include variations of FES uptake in different ER+ breast cancer diseases and exclusion of posterior breast tissue near the chest wall and the axillary regions. In evaluating response to treatment, other uptake metrics adjunct to SUV_max_ may be considered to describe the overall ER status before and after treatment. Further studies involving larger numbers of patients are needed to validate our initial observations.

## Methods

### Ethics statement

Six patients with biopsy-confirmed >90% ER+/HER2− breast cancer were recruited to participate in an imaging study with dbPET (MAMMI, General Equipment and Medical Imaging SA (OncoVision), Valencia, Spain). The dbPET imaging study was a HIPPA-compliant study protocol that was reviewed by the institutional review board and approved by the Committee of Human Research under the institution Human Research Protection Program. The use of FES for human imaging was approved by the Radioactive Drug Research Committee. A written informed consent was provided by the patient to participate.

### Patient characteristics

To limit patient’s radiation exposure, a separate whole-body FES-PET/CT imaging was not performed. All patients underwent physical examination for regional adenopathy and breast MRI. Patients with adenopathy identified by clinical examination or ultrasound evaluation underwent needle biopsy of the nodes. Patients with clinical stage III disease underwent whole-body FDG-PET/CT or chest/abdomen pelvis CT. Staging was obtained per requirements of additional clinical trials for some patients. Other patients received a whole-body single photon emission tomography (SPECT) with [^99m^Tc] hydoxydiphosphonate for bone metastases.

### DbPET imaging

DbPET was performed with a low dose of FES ranging 4.94–5.38 mCi (median, 4.99 mCi) at 45 min post-injection. Patients were scanned in the prone position with a single breast positioned down through the aperture inside the detector ring, which can be translated axially to image the entire breast from inferior to superior at approximately 15 min per breast. FES dose at the time of injection and time of imaging was recorded. A follow-up dbPET scan was performed in two patients after 2 months of endocrine treatment (patient #1) and three weekly cycles of chemotherapy without endocrine treatment (patient #3). Same imaging procedure and tracer dose was repeated as performed at baseline. DbPET images were reconstructed in 3D using the manufacturer-provided maximum likelihood expectation maximization (MLEM)^29^ algorithm with 16 iterations. All images were corrected for attenuation through image segmentation, scatter, and decay.^30^ Tumor volume-of-interest (VOI) encompassing the entire abnormal tissue volume was calculated using an SUV cutoff of 2.5. Subsequent analyses of tumor FES uptake were performed by (1) calculating maximum and mean SUVs normalized by body weight (SUV_max_, SUV_mean_);^31^ (2) maximum and mean SUVs normalized by lean body mass (SUL_max_, SUL_mean_); (3) examining the tumor-to-nontumor ratio by placing VOIs on tumor and normal breast tissue within the field of view; and (4) total FES uptake volume.

### Reporting Summary

Further information on experimental design is available in the Nature Research Reporting Summary linked to this article.

## Supplementary information

Reporting Summary

## Data Availability

The data generated and analyzed during this study are described in the following data record:^32^ 10.6084/m9.figshare.7642844. As described in the data record, DICOM image files and FES-dbPET imaging results are available from the authors on request. Aggregate level data are available within the article tables.

## Supplementary information

**Supplementary information** accompanies the paper on the *npj Breast Cancer* website (10.1038/s41523-019-0107-9).
